# Deep learning model for classification and bioactivity prediction of essential oil-producing plants from Egypt

**DOI:** 10.1038/s41598-020-78449-1

**Published:** 2020-12-07

**Authors:** Noha E. El-Attar, Mohamed K. Hassan, Othman A. Alghamdi, Wael A. Awad

**Affiliations:** 1 Information Systems Department, Faculty of Computers and Artificial Intelligence, Banha University, Banha, Egypt; 2 Biotechnology Program, Zoology Department, Faculty of Science, Port Said University, Port Said, Egypt; 3 Department of Biological Sciences, Collage of Science, University of Jeddah, Jeddah, Kingdom of Saudi Arabia; 4 Mathematics and Computer Science Department, Faculty of Science, Port Said University, Port Said, Egypt; 5 Computer Science Department, Faculty of Computers and Information, Damietta University, Damietta, Egypt

**Keywords:** Biochemistry, Computational biology and bioinformatics, Plant sciences

## Abstract

Reliance on deep learning techniques has become an important trend in several science domains including biological science, due to its proven efficiency in manipulating big data that are often characterized by their non-linear processes and complicated relationships. In this study, Convolutional Neural Networks (CNN) has been recruited, as one of the deep learning techniques, to be used in classifying and predicting the biological activities of the essential oil-producing plant/s through their chemical compositions. The model is established based on the available chemical composition’s information of a set of endemic Egyptian plants and their biological activities. Another type of machine learning algorithms, Multiclass Neural Network (MNN), has been applied on the same Essential Oils (EO) dataset. This aims to fairly evaluate the performance of the proposed CNN model. The recorded accuracy in the testing process for both CNN and MNN is 98.13% and 81.88%, respectively. Finally, the CNN technique has been adopted as a reliable model for classifying and predicting the bioactivities of the Egyptian EO-containing plants. The overall accuracy for the final prediction process is reported as approximately 97%. Hereby, the proposed deep learning model could be utilized as an efficient model in predicting the bioactivities of, at least Egyptian, EOs-producing plants.

## Introduction

Recently, Artificial Intelligence (AI) has become one of the vigorous science that infiltrated a huge number of modern life issues such as chemical engineering, water treatment, and biological domain like genomic and proteomic studies which are especially characterized by complicated and non-linear processes^1^. Deep learning is one of the most promising branches of artificial intelligence with proven power in taking the raw features extracted from the extremely large data sets, such as the data produced from genomics, chemistry, and pharmaceutical laboratories. Processing these data result in inferred patterns and training process-based predictive models^2^.

Essential Oils (EOs) are biologically effective organic compounds extracted from different parts of the aromatic plants such as flowers, leaves, and barks to name a few^3^. Due to their wide range of biological activities, these natural products are widely used in complementary and alternative medicine (CAM). Replacing the inorganic chemistry by natural alternatives is still hot topic in recent biological area of research. This is because the inorganic chemical products may bereave harmful influences when used in health-related industries, such as medicine, pharmaceutics, cosmetics, food, and beverages. Therefore, the modern researches head to find the alternative natural products, including EOs, due to their greater ability to adapt to alive organs of the human body with, sometimes, limited side effects^4^.

Generally, the bioactivity of the EO-producing plant depends on the chemical structure and EOs content, which determine the overall bioactivity of such plant. EOs are, in fact, composed of different combinations of low nuclear weight natural blends with complete organic locomotion. According to their structure, these dynamic blends can be categorized into some significant pools (e.g. hydrocarbons, oxygenated mixtures, and sulfur or conceivably nitrogen). These mixes’ pools are the mystery key for the biological activity of each EO^3^.

The biological activities of the EOs may include antiseptics, antimicrobials, antifungals, antioxidant, antitumor, antivirals, and/or anti-inflammatories. Moreover, these activities vary according to the chemical constitution, which may differ from plant to another according to their geographic location, agriculture conditions, climatic or seasonal changes^4^. Noteworthy, the evaluation of the EOs' bioactivities cannot be constantly credited to one single compound in the EOs mixture. The genuine connections between the EO’s cocktail and its biological activities are highly non-linear, especially when considered across variable pools of chemical structures. Hence, reliance on traditional techniques in predicting the biological impact for such activity data with this variety of structures is a troublesome issue^5^. Therefore, developing a deep learning-based computational model to categorize and predict the biological activities of EOs-producing plants based on their chemical construction’s variations, without recourse to in-vitro experiments, could save time and cost.

Machine learning (ML) algorithms, especially Artificial Neural Networks (ANN), have been proposed to contribute in solving several biological issues in the recent decades^6^. ANN, in general, can be depicted as a numerical model of a particular structure, comprising of some of the single processing components (i.e. nodes and neurons), constructed between inter-connected layers. Each entire layer is mainly composed of hidden neurons which are responsible for transforming the input values and sending the outputs to the other associated neurons^1^. Recently, due to the expansion of the biological information, the fully connected neural network would have a huge number of parameters, which needs full processing inside the network layers to deliver the desired output. Deep learning approaches have proven their efficiency in the applications whose data are characterized by their large quantities, high dimensionality, and highly structured. Thus, deep learning approaches are widely used in image processing due to the nature of the image which contains many thousands of variables (pixels) that can be clearly grouped into well- defined objects^6^. However, deep learning approaches are no longer limited to image processing domain, where it is recently considered an attractive solution for some types of text classification such as DNA sequences classification problems^7^. From this standpoint, deep learning can be an efficient learning approach for dealing with the complex composition of the chemical compounds and their interrelationships with biological activities.

**Figure 1:**
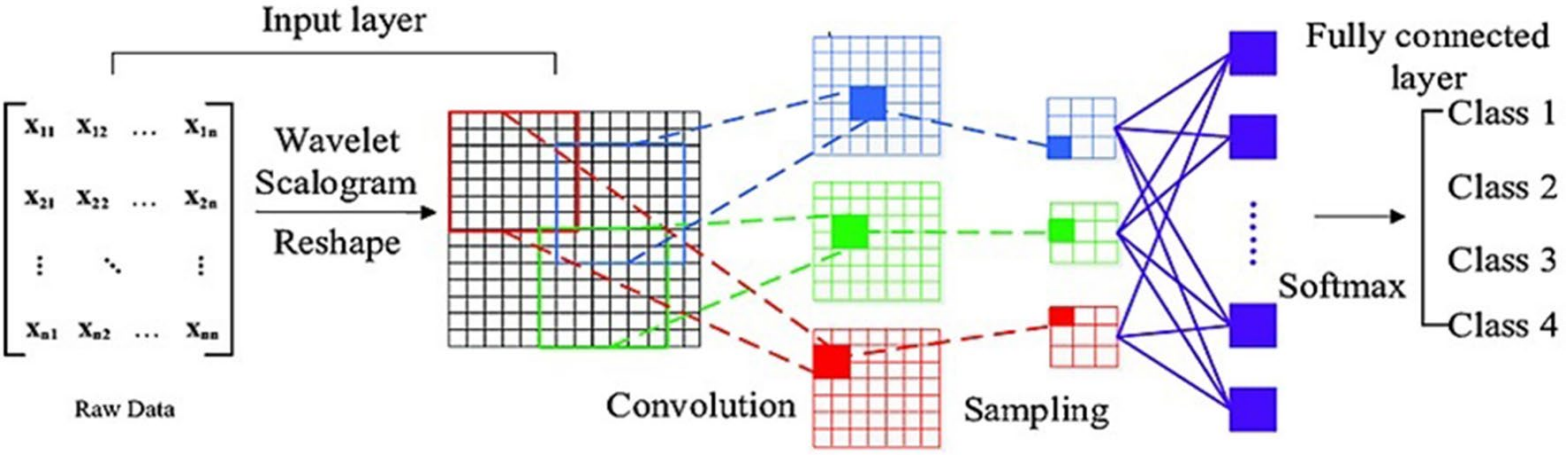
Standard Convolutional Neural Network Architecture usually consists of an input layer, convolution layer, Max pooling and the final fully-connected neural network layer which gives the output based on the activation function^9^.

One of the efficient models for the deep learning is the Convolutional Neural Network (CNN). The CNN is characterized by two novel types of layers: *convolution and pooling layers*. These layers are based on using filters to convolve the range of the input data to a smaller range, detecting important or specific parts within this range^8^. The CNN usually consists of Input Layer , Convolution Layer (i.e. produces a matrix of dimension smaller than the input matrix), ReLU or Rectified Linear Unit . ReLU is mathematically expressed as Max(0,* x*) (i.e. it means that any number below 0 is converted to 0, while any positive number is allowed to pass as it is), Max pooling (i.e. passes the maximum value from amongst a small collection of elements of the incoming matrix to the output) and the final output layer (i.e. a fully-connected neural network layer, which makes the output based on the activation function), as shown in Fig. 1^9^.

This study seeks to classify and predict the biological activities of the Egyptian essential oil-producing plants based on their EOs content as an experimental case study. The classification is implemented based on two algorithms: Multiclass Neural Network (MNN) and Convolutional Neural Network (CNN), to evaluate the efficiency of both machine and deep learning techniques. The effective algorithm is adopted in developing a biological activity prediction model EOs-producing cases cultivated in Egypt. The research skills in this study are organized as follow; first, presenting the results obtained from implementing the two algorithms; MNN as a machine learning algorithm and CNN as a deep learning algorithm, when applied on the Egyptian essential oils dataset. Next, these results were discussed and evaluated for the existing dataset and the new untested datasets. Finally the methodologies followed in this work are discussed.

## Results

### Using MNN- and CNN-based algorithms to classify the plants’ EOs bioactivity

The results recorded from the classification process for both MNN and CNN algorithms are summarized in Tables 1 and 2. In the CNN and MNN algorithms, the training processes show an overall accuracy of 100% and 99.2%, of correct classification of essential oils activity, respectively. Whilst, for the testing stage, the overall accuracy achieved by CNN and MNN is 98.13% and 81.88%, respectively.

**Table 1:** The confusion matrix values for MNN and CNN algorithms in testing stage.

Bioactivity class Classifiers	True positive		True negative		False positive		False negative	
	MNN	CNN	MNN	CNN	MNN	CNN	MNN	CNN
Antiviral	50%	97.7%	94.4%	98.2%	5.6%	1.8%	50%	2.3%
Antiwormal	100%	100%	98.3%	98.5%	1.7%	1.5%	0	0%
Anti-inflammatory	90%	100%	80%	100%	20%	0%	10%	0%
Anticancer	85.7%	94%	96.2%	93.3%	3.8%	6.7%	14.3%	6%
Antioxidant	75.9%	98.4%	80.6%	98.6%	19.4%	1.4%	24.1%	1.6%
Antimicrobial	78.8%	97.4%	66.7%	97.3%	33.3%	2.7%	21.2%	2.6%
Antifungal	81.3%	98%	86.4%	99.2%	13.6%	0.8%	18.8%	2%
Cytotoxic activity	58.3%	96.6%	93.8%	98.7%	6.3%	1.3%	41.7%	3.4%

**Table 2:** The accuracy and relevance metrics for MNN and CNN algorithms in testing stage.

Bioactivity class Metrics	Average accuracy		Precision		Recall		F1 score	
	MNN	CNN	MNN	CNN	MNN	CNN	MNN	CNN
Antiviral	0.72	0.99	0.9	0.98	0.5	0.99	0.64	0.99
Antiwormal	0.99	0.99	0.98	0.99	1	1	0.99	0.99
Anti-inflammatory	0.85	1	0.82	1	0.9	1	0.86	1
Anticancer	0.9	0.94	0.96	0.93	0.86	0.94	0.9	0.94
Antioxidant	0.78	0.99	0.8	0.99	0.76	0.98	0.78	0.98
Antimicrobial	0.73	0.97	0.7	0.97	0.79	0.97	0.74	0.97
Antifungal	0.84	0.99	0.86	0.99	0.812	0.98	0.83	0.99
Cytotoxic activity	0.76	0.98	0.9	0.99	0.58	0.97	0.71	0.98

The confusion matrix is the most suitable way to validate the classification performance. Here, the confusion matrix of the MNN and CNN classification is presented in Table 1. It consists of four outcomes of binary classifiers: True Positive, False Positive, True Negative, and False Negative. Also, accuracy, precision, recall, and F1 score are different metrics that are used for evaluating the classification efficiency based on the values of the confusion matrix^10^. The four metrics for the bioactivities classes have been calculated and documented in Table 2.

### Using a CNN-based algorithms to build a prediction model for the EOs bioactivity

**Figure 2:**
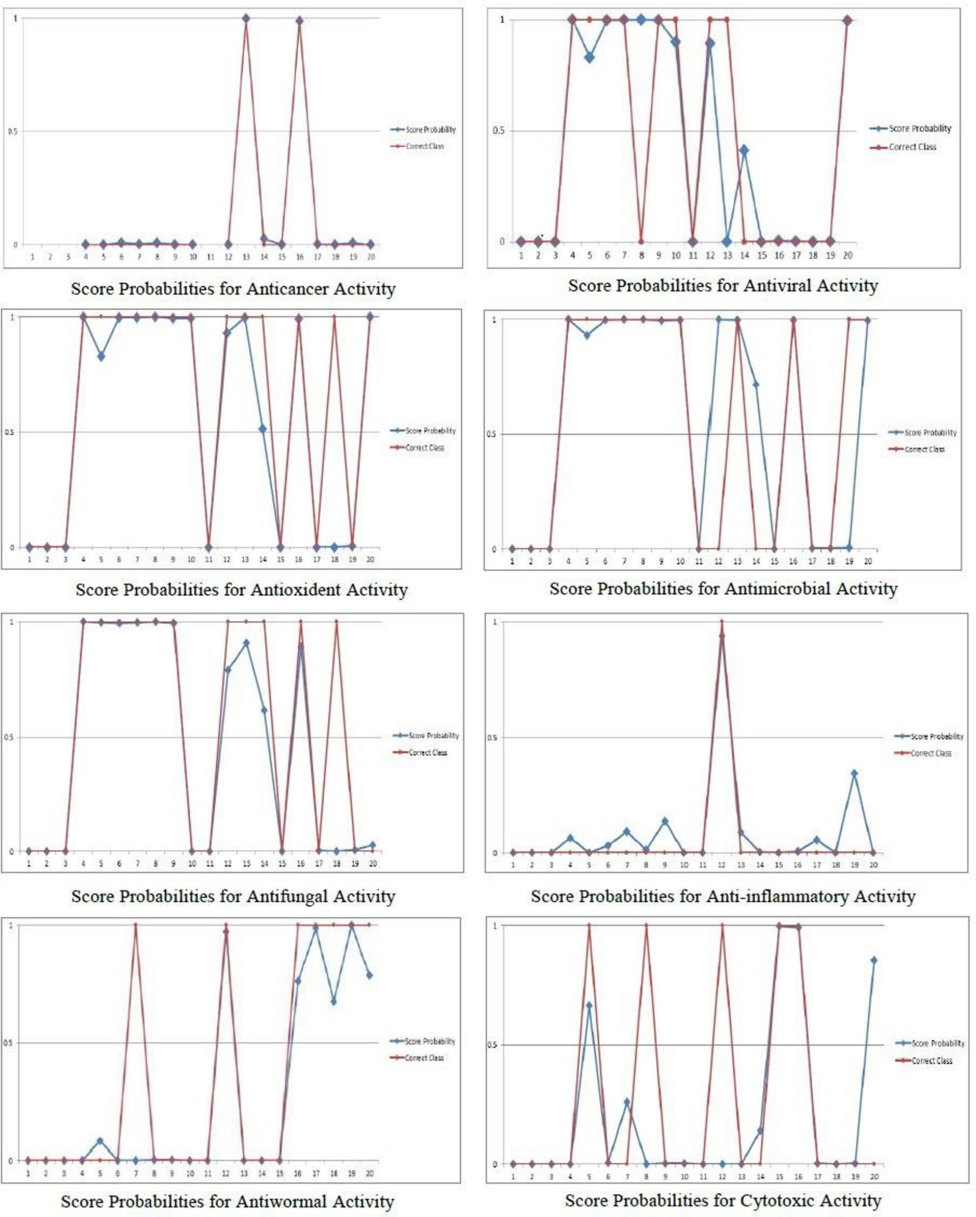
The discrimination outcome of the developed CNN for predicting the biological activities for a new essential oils’ dataset.

In accordance with the completion of the classification process, the CNN algorithm show high accuracy for the training and testing processes. This is due to its capabilities in dealing with the huge number of data and focusing on the high impact features in the dataset. Thus, the biological activity prediction model has been built based on the CNN proposed algorithm. The overall accuracy for predicting the biological activities for previously unknown Egyptian essential oils components has been recorded as approximately 97%. The concluded metrics values and the discrimination outcomes of the proposed CNN prediction model are reported in Tables 3 and 4, and in Fig. 2.

**Table 3:** The confusion matrix values for the predicting model.

Bioactivity class Classifiers	True positive	True negative	False positive	False negative
Antiviral	98%	96.3%	3.7%	2%
Antiwormal	100%	98.3%	1.7%	0%
Anti-inflammatory	100%	100%	0%	0%
Anticancer	97%	94.7%	5.3%	3%
Antioxidant	100%	95%	5%	0%
Antimicrobial	94%	93.5%	6.5%	6%
Antifungal	100%	96.6%	3.4%	0%
Cytotoxic activity	94%	93.1%	6.9%	6%

**Table 4:** The accuracy and relevance metrics for the predicting model.

Bioactivity class Metrics	Average accuracy	Precision	Recall	F1 score
Antiviral	0.97	0.96	0.98	0.97
Antiwormal	0.99	0.98	1	0.99
Anti-inflammatory	1	1	1	1
Anticancer	0.96	0.95	0.97	0.96
Antioxidant	0.98	0.95	1	0.98
Antimicrobial	0.94	0.94	0.94	0.94
Antifungal	0.98	0.97	1	0.98
Cytotoxic activity	0.94	0.93	0.94	0.94

## Discussion

Adopting the idea of using AI, especially machine learning and deep learning algorithms, has become a vital topic in medical and biological problem solving. EOs are considered as one of the well-known natural products which have medical-relevant use and biological-defense activities against several types of viruses, bacteria, and cancer^11^. However, the lack of the complete information about their intrinsic chemical variability and their function makes it difficult to confirm its consistent activities^4^.

Many researchers have adopted the AI in EO's researches aiming to reduce the in-vitro procedures and to make reasonable predications of the experiment results. For instance, Ragno et al. (2020) have developed an unsupervised machine learning algorithm to cluster the EOs and to identify the EOs that have strong ability in inhibiting bacterial growth of all bacterial strains^12^. Moreover, Artini et al. (2018) have exhibited a binary classification model based on ML to classify the essential oils activities from different Mediterranean plants against pseudomonas aeruginosa^13^. Similarly, Daynac et al. (2015) have used the fast artificial neural networks (FNN) to predict the antimicrobial activity of 49 EOs against four types of pathogens. The FNN algorithm predicted more than 70% of the antimicrobial activities within a 10 mm maximum error range^4^.

Egypt is one of the biggest countries in the world in exporting the high-quality raw material of more than 150 medicinal and aromatic plants. The variety and massiveness of aromatic plant species in Egypt, stemming from the climatic conditions in its environment, may stimulate the accumulation of high concentrated secondary metabolites. This makes the Egyptian aromatic plants are considered between the most promising sources for many biologically active compounds^14^. Thus, there is an imperious need to extend the scientific knowledge base of the aromatic species in Egypt by the modern AI methodologies using machine and deep learning.

The experimental study, here, is applied on the Egyptian case for the essential oils. The data were collected from several peer researches article including in-vitro experiments. For each case, the in-vitro experiment may only focus in analyzing the chemical composition of the EO, or apply one or more of EOs on a specific infection type (e.g. bacteria, viruses, or cancer). This encouraged the authors, here, to experiment the machine and deep learning algorithms in predicting the biological activities for some of the Egyptian essential oils-producing plants. The training and testing processes are conducted on a novel dataset of EOs-producing plants from Egypt, that are collected and manipulated by authors from peer reviewed scientific researches^11–55^. The resulting dataset consists of a sample of one hundred and twenty (120) plants. The total chemical compounds extracted from this dataset are 573 compounds. In the proposed experimental case, the biological activities of the essential oils are classified according to eight categories of bio-activities (anticancer, antioxidant, antimicrobial, antifungal, antiviral, anti-wormal, anti-inflammatory, and cytotoxic activities).

**Figure 3:**
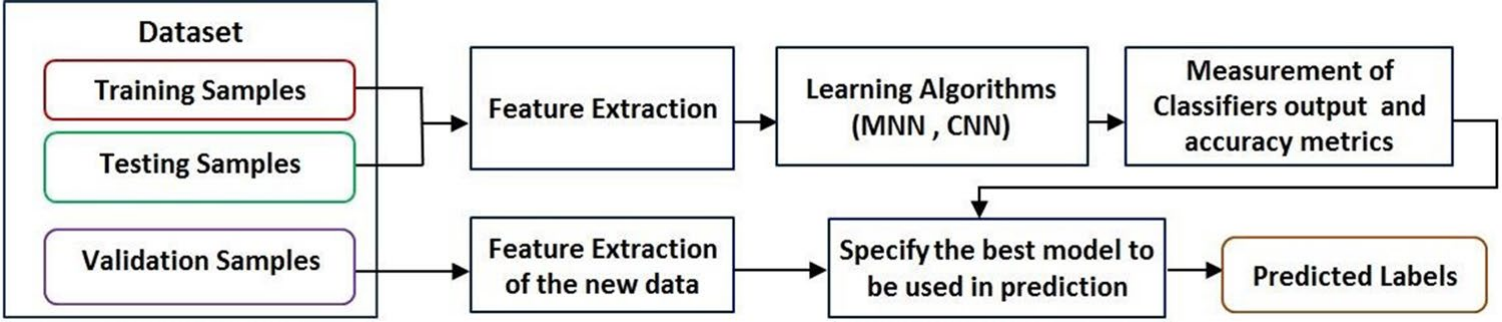
An inclusive flowchart for the proposed methodologies stages.

**Figure 4:**
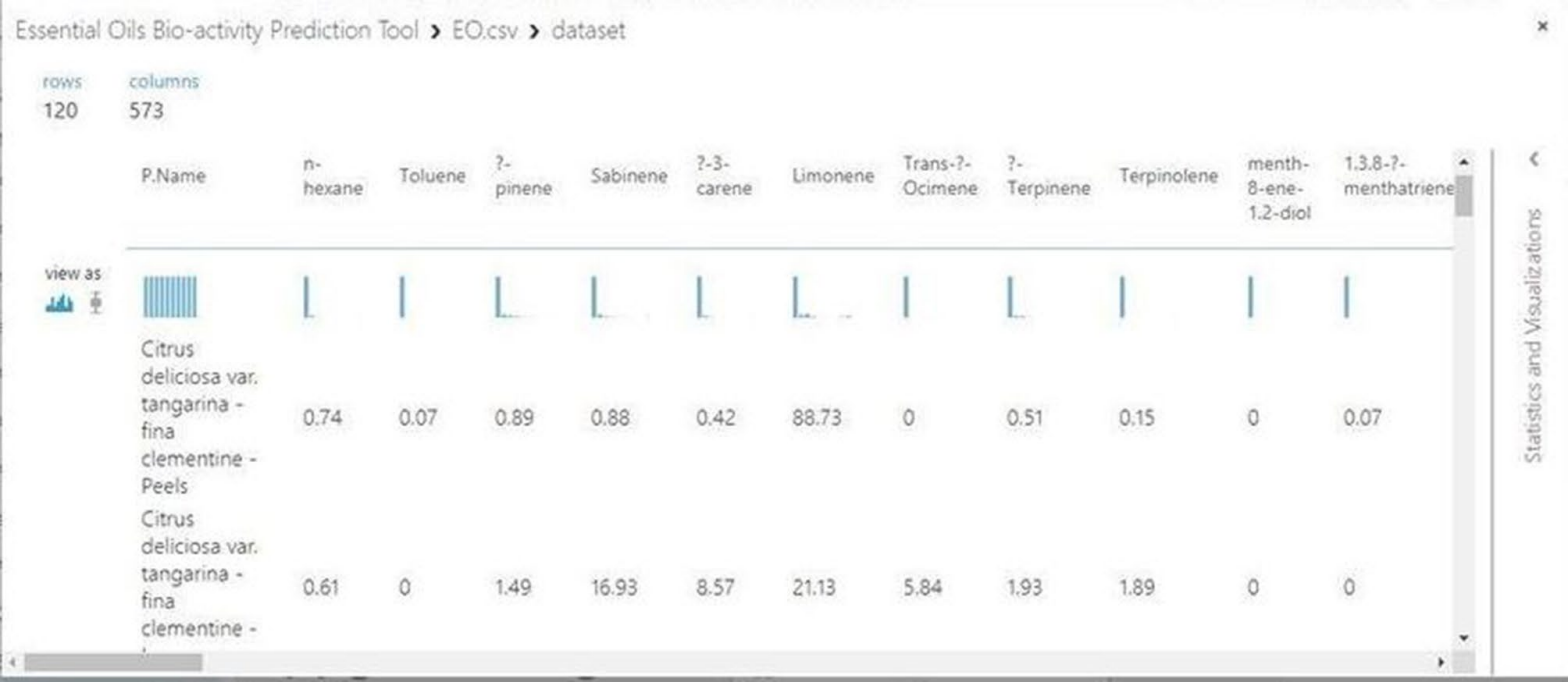
Sample for the Essential oils dataset imported by Azure. The Picture is taken from the Azure machine learning studio (classic) platform, https://studio.azureml.net/.

In order to classify the EO's according to their biological activities, two classification models rely on the supervised learning are constructed; the first one is based on the Multiclass Neural Network (MNN) (i.e. the Multiclass N.N module in Azure). The second model depends on the Convolutional Neural Network (CNN) and it has been implemented by Python and executed as a module on the open source Azure Machine learning studio^56^. Figure 3 presents an inclusive flowchart for the proposed methodology stages. The two proposed supervised learning algorithms are applied on nearly 68,760 values that represent the percentage of chemical compounds concentrations in the EO’s dataset on study. The training process for both MNN and CNN algorithms is done on 60% of the dataset, whilst the remaining 40% is divided between the testing and the prediction processes. That means the actual size for the training, testing, and validation processes are 45,840, 11,460, and 11,460 values, respectively. Figure 4 shows a sample for the EOs dataset stored in azure format.

In the proposed MNN algorithm, the training model runs a sequence of binary classifiers and trains each to decide a separate classification outcome according to the *softmax* activation function results. The architecture of the proposed MNN is a fully connected layers network with one hidden layer contains 50 nodes, and an output layer with 8 nodes each one represents an output class. The outputs of the hidden layer $O{h}_{i}$ and the output layer ${O}_{k}$ are calculated by Eq. 1 and 3 respectively^57^.

1 $$O{h}_{i}=\frac{1}{1+{e}^{{-zh}_{i}}} \forall i=1,\dots \dots , 50$$

where

2 $${Zh}_{j}={\sum }_{j=1}^{n}{x}_{j}.{w}_{j}+b \forall j=1,\dots \dots ,n$$

3 $${O}_{k}=\frac{{e}^{{Oh}_{k}}}{{\sum }_{k=1}^{L}{e}^{{Oh}_{k}}}\forall k=1\dots \dots 8$$

where

4 $${Oh}_{k}={\sum }_{i=1}^{50}{oh}_{i}.{w}_{i}$$

In general, MNN proves its efficiency when the total amount of data is limited. Hereby, more significantly, another efficient classification model based on the CNN is created. The proposed CNN is developed in such a way to handle the ambiguity and inconsistency that appeared in the chemical compositions values which could not be fully treated with the MNN. One of the mysterious characteristics in the essential oil(s) is that, they may contain some chemical compounds that have no biological influence activity, and this may be due to the inconsistency among these compounds. Thus, a number of compounds/oils that have no effect in a certain EO’s pool may be found, but they may show a clear influence if they are found with other compounds in another EO pool^58^.

The problem in this study is categorized as multi-label classification problem, where the essential oils can have multiple activities (i.e. outputs) at the same time. Thus, in regards with the proposed dataset, the CNN could produce eight output labels for each essential oil (i.e. the eight biological activities), where an essential oil may have all of these activities or some of them. The output labels have been encoded in the form of a one-hot encoded vector with multiple ones in it, as a special form of the one-hot encoding method. For instance, the Essential oil “*Pluchea dioscoridis*” is known with its activities as antimicrobial, antioxidant, and anticancer, so its label will be [0,0,0,1,1,1,0,0] for the target vector [*Antiviral, Antiwormal, Anti-inflammatory, Anticancer, Antioxidant, Antimicrobial, Antifungal, and Cytotoxic Activity*]*.* Table 5 shows a sample of the CNN training results on the dataset where it documents the score probabilities that outcome from the sigmoid activation function. These score values refer to predicted biological activities labels for a number of Essential oils.

**Table 5:** A sample of the Score Probabilities for the CNN Training Process.

Eos name Score probabilities	Antiviral	Antiwormal	Anti-inflammatory	Anticancer	Antioxidant	Antimicrobial	Antifungal	Cytotoxic activity
*Citrus deliciosa* var. *tangarina*—fina clementine—peels	0.9888	0.0014	0.0032	0.0001	0.9854	0.9981	0.9983	0.9966
*Citrus deliciosa* var. *tangarina*—fina clementine—leaves	0.9999	0.9986	0.0005	0.0003	0.9992	0.9984	0.9983	0.0013
*Citrus deliciosa* var. *tangarina*—Nour Clementine—peels	0.9465	0.0022	0.3243	0.0002	0.9894	0.9854	0.9684	0.7711
*Citrus deliciosa* var. *tangarina*—Nour Clementine—leaves	0.9999	0.0013	0.0641	0.0002	0.9995	0.9997	0.9996	0.0003
*Citrus deliciosa* var. *tangarina*—Spinosa Clementine—peels	0.8294	0.0795	0.0001	0.0003	0.8291	0.9294	0.9993	0.6654
*Citrus deliciosa* var. *tangarina*—Spinosa Clementine—leaves	0.9977	0.0015	0.0320	0.0084	0.9968	0.9982	0.9961	0.0049
*Citrus deliciosa* var. *tangarina*—Thornless Clementine—peels	0.9991	0.0007	0.0931	0.0034	0.9987	0.9999	0.998	0.2611
*Citrus deliciosa* var. *tangarina*—Thornless Clementine—leaves	0.9999	0.0020	0.0134	0.0082	0.9999	0.9998	0.9996	0.0007
*Callistemon comboynensis*	0.9975	0.0037	0.1375	0.0011	0.9947	0.9955	0.9945	0.0031
*Cupressus sempervirens* L *Semperuirens* L	0.8982	0.0011	0.0004	0.0012	0.9947	0.9967	0.0002	0.0035
*Cuminum cyminum*	0.8923	0.9747	0.9384	0.0005	0.9297	0.9997	0.7909	0.0001
*Ocimum basilicum *L. (sinai )	0.0004	0.0005	0.0883	0.9977	0.9991	0.9990	0.9089	0.0003
*Tagetes minuta* L	0.4107	0.0011	0.0034	0.0253	0.5146	0.7167	0.6179	0.1418
*Achillea fragrantissima*	0.0004	0.0003	0.0003	0.0007	0.0008	0.0013	0.0001	0.9985
*Pluchea dioscoridis*	0.0052	0.7622	0.0054	0.9863	0.9931	0.9991	0.8932	0.9915
*Myrtus communis*—leaves	0.0022	0.9887	0.0554	0.0018	0.0003	0.0021	0.0023	0.0028
*Myrtus communis—*fruits	0.0007	0.6754	0.0001	0.0005	0.0013	0.0023	0.0011	0.0004
*Eugenia supraxillaris—*leaves	0.001	0.9998	0.3451	0.0075	0.0050	0.0075	0.0065	0.0032
*Eugenia supraxillaris—*fruits	0.9955	0.7865	0.0003	0.0003	0.9995	0.9934	0.0273	0.8539

## Conclusion

Developing a computational model based on deep learning for classifying and predicting the EOs' biological activities without resorting to in-vitro experiments is the challenge of this study.

Due to the efficacy of the EOs as antiseptics, antimicrobials, antifungals, antioxidant, antitumor, antivirals, and anti-inflammatories, they have a great attention from the health concerns industries, such as medicine, pharmaceutics, cosmetics, and others. However, there is a significant challenge in deciding the relevance between the chemical compounds that form the EO and its biological activities through the traditional in-vitro experiments. In this study, two classification models are implemented to classify and predict the biological activities of 120 types of Egyptian essential oils as an experimental study case. This experiment has been implemented based on two types of supervised learning algorithms, Multiclass Neural Network and Convolutional Neural Network in order to evaluate the efficiency of both machine and deep learning techniques. The comparison between the accuracy and relevance metrics for both MNN and CNN algorithms in the testing stage showed that the CNN outperformed the MNN as it scored an accuracy rate 98.13%, while the MNN recorded 81.88%.

## Method

**Figure 5:**
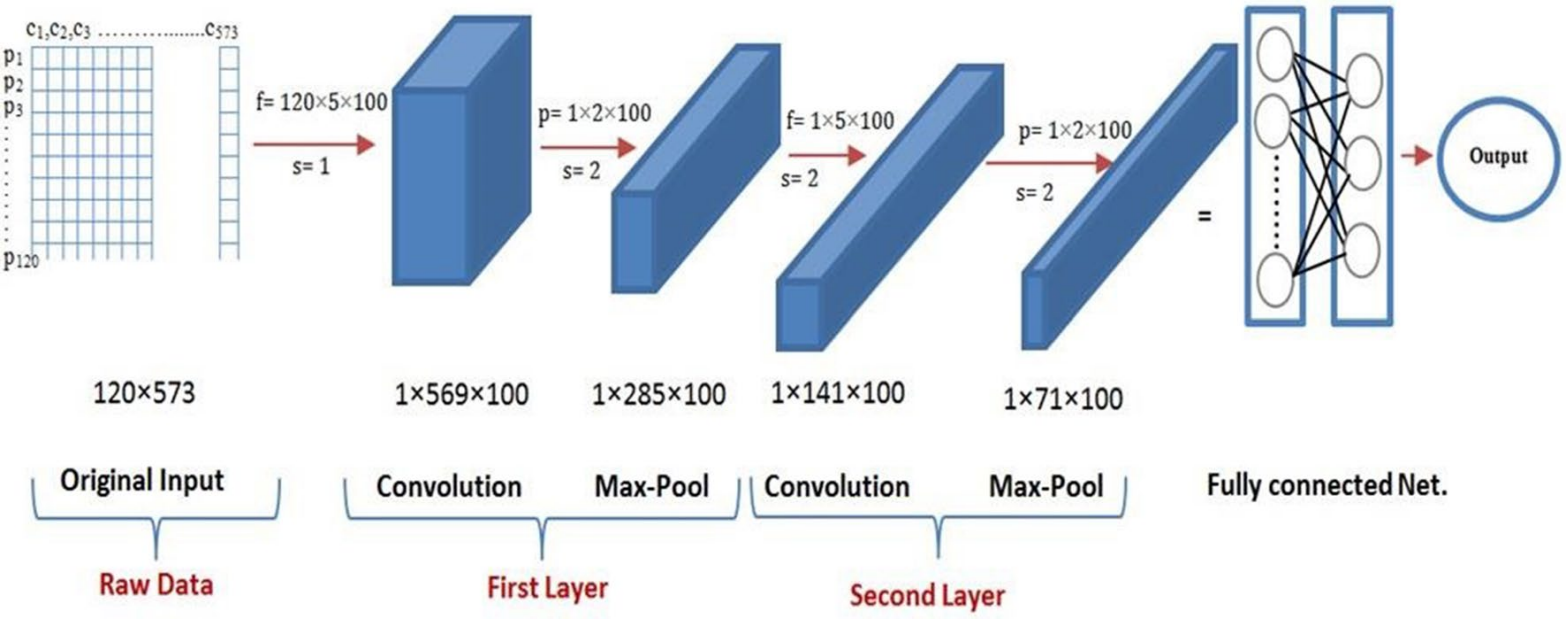
The Architecture of the Proposed CNN, it mainly begins with an original input matrix represents the essential oils data. The internal network schema based on two layers of convolution and pooling. The output of these layers feeds a fully connected feed forward neural network that uses a sigmoid activation function to find the appropriate output.

The model of the CNN, which is utilized in this experiment, comprises of a three-layers fully connected network with two convolution layers and two pooling layers followed by one hidden layer as shown in Fig. 5. The input information for the proposed CNN is a 2D matrix of [120 × 573] that represents “the number of essential oils and the numbers of their chemical compounds, respectively”. The following sequential layers start with a convolution layer which applies a convolutional process to the input matrix. The output of each node in the convolution layer is the result of a convolution operation by each filter. The next layer is the pooling layer which is used to compress information and generalize features to reduce the overfitting of the training data. In this experiment, the local max-pooling, which produces the maximum value from small divided regions in the input matrix, is used. The convolution and pooling outputs are calculated by the following equation;

5 $${O}_{k}^{l}=\sum {a}_{k}^{l}*{w}_{k}^{l}+{b}_{k}^{l}$$

where ${O}_{k}^{l}$ is the output vector of the *l*th layer with *k*th kernel, ${a}_{k}^{l}$ is the input vector, ${w}_{k}^{l}$ is the weight of the convolution/pooling filter, and ${b}_{k}^{l}$ is the bias coefficient.

During the CNN learning process, seven hyper-parameters are tuned (convolution filter size (*k*), number of filters (*f*), stride size (*s*), pooling size (*p*), number of nodes in the hidden layer, and the activation function). The numbers of iterations for the learning process are specified around 100. Table 6 displays the values of the assumed hyper-parameters and the output layer dimensions. The individual layer dimension can be calculated by^8^;

**Table 6:** Description of CNN layers with the hyper-parameters values.

Layers	Filter size	Stride	Output size	Output Dense
Input	–	–	120 × 573	68,760
1st convolution layer	120 × 5 × 100	1	1 × 569 × 100	56,900
1st max pooling layer	1 × 2 × 100	2	1 × 285 × 100	28,500
2nd convolution layer	1 × 5 × 100	2	1 × 141 × 100	14,100
2nd max pooling layer	1 × 2 × 100	2	1 × 71 × 100	7100

6 $${n}^{l}\times {m}^{l}=\left[\left[\frac{{n}^{l-1}-{f}^{l}}{{s}^{l}}\right]+1\right]\times \left[\left[\frac{{m}^{l-1}-{f}^{l}}{{s}^{l}}\right]+1\right]$$

Finally, the sequence of the convolutional and pooling layers ends with a fully connected feed-forward neural layer that uses a “sigmoid” activation function. The sigmoid function is selected in the proposed CNN implementation because its function depends mainly on converting each score of the final node to a probability value between 0 to 1, independent of what the other scores are. So, the input could be classified into multiple independent classes (Suppl Information).

## Supplementary Information

Supplementary Legends.

Supplementary Dataset.

## Data Availability

The complete data set is available in the supplementary file (sup-file). The dataset is formatted to be suitable to be processed by Azure ML modules.
