# Supplementary material for: Deep learning model for classification and bioactivity prediction of essential oil-producing plants from Egypt
Source: Sci Rep. 2020 Dec 7;10:21349. doi: 10.1038/s41598-020-78449-1 (PMC7721748; doi:10.1038/s41598-020-78449-1)
Supplement: Supplementary file 1 — Supplementary Legends. [file 41598_2020_78449_MOESM1_ESM.docx]

**Supplementary File Legend**

The supp_file.csv contains the complete data set that is used in the presented implementation. The dataset is formatted to be suitable to be processed by Azure ML modules.
